# Screening of Monoamine Oxidase Inhibitors from Seeds of *Nigella glandulifera* Freyn et Sint. by Ligand Fishing and Their Neuroprotective Activity

**DOI:** 10.3390/plants12040882

**Published:** 2023-02-15

**Authors:** Emmanuel Ayodeji Ayeni, Chao Ma, Yikao Hu, Xiaolin Bai, Yongmei Zhang, Xun Liao

**Affiliations:** 1 Chengdu Institute of Biology, Chinese Academy of Sciences, Chengdu 610041, China; 2 University of Chinese Academy of Sciences, Beijing 100049, China; 3 Phytochemistry Laboratory, Tibet Plateau Institute of Biology, Lhasa 850001, China

**Keywords:** *Nigella glandulifera*, ligand fishing, neuroprotection, monoamine oxidase-B inhibitor, Parkinson’s disease

## Abstract

*Nigella glandulifera* is a traditional medicinal plant used to treat seizures, insomnia, and mental disorders among the Tibetan and Xinjiang people of China. Recent pharmacological research indicates that the seeds of this plant have a neuroprotective effect; however, the chemical components responsible for this effect are unknown. Monoamine oxidase B (MAO-B) has been recognized as a target for developing anti-Parkinson’s disease drugs. In this work, MAO-B functionalized magnetic nanoparticles were used to enrich the enzyme’s ligands in extracts of *N. glandulifera* seeds for rapid screening of MAO-B inhibitors coupled with HPLC-MS. Tauroside E and thymoquinone were found to inhibit the enzyme with IC_50_ values of 35.85 μM and 25.54 μM, respectively. Both compounds exhibited neuroprotective effects on 6-OHDA-induced PC-12 cells by increasing the cell viability to 52% and 58%, respectively, compared to 50% of the injured cells. Finally, molecular docking indicated strong interactions of both inhibitors with the enzyme. This work shows that MAO-B functionalized magnetic nanoparticles are effective for rapid screening of anti-PD inhibitors from complex herbal mixtures and, at the same time, shows the promising potential of this plant’s seeds in developing anti-PD drugs.

## 1. Introduction

Parkinson’s disease (PD) is the second most common neurodegenerative disorder and constitutes a societal health problem, especially among older people around the world [1]. PD affects motor and non-motor neurons and exhibits various symptoms such as muscle tremors, rigidity, bradykinesia, loss of stability, and body gaits [2]. Pathological characteristics of PD include the aggregation of alpha-synuclein in the intraneuronal cytoplasm, known as Lewy bodies, and the progressive damage of dopamine neurons in the substantia nigra pars compacta [3]. To date, there is no known cure for Parkinson’s disease. The treatments for this disease, either medications or surgical procedures, are useful only for controlling the symptoms. Several influencing factors such as oxidative stress, mitochondrial dysfunction, genetics, age, environment, gender, and ethnicity have been highlighted as possible predisposing factors to Parkinson’s disease and other related neuronal disorders [4,5]. Globally, there is an increasing number of people living with Parkinson’s disease, as well as several other neurodegenerative diseases. For instance, in China, it is projected that PD patients may increase to approximately 5 million within a decade, thus accounting for a huge proportion of people over 60 years old [6,7,8]. Further, the cost of anti-PD medication exceeds the average person’s economic capacity and creates a huge burden of care [9].

Monoamine oxidases (MAO) is a group of mitochondrial enzymes that are flavin adenine dinucleotide-dependent and catalyze the oxidative deamination of structurally diverse amines. These enzymes are present in two isoform types (MAO-A and MAO-B). In humans, there is an association between MAO-B activity and age, in which the presence of MAO-B is higher in certain neurodegenerative diseases such as Parkinson’s disease [10]. Pharmacological studies have shown that MAO-B plays a significant role in the development of Parkinson’s disease [11,12], and inhibitors of MAO-B help treat PD by upregulating levels of dopamine, a crucial neurotransmitter in PD pathogenesis [13,14]. MAO-B inhibitors, such as selegiline, rasagiline, and safinamide, have been widely used in treating PD clinically. However, long-term usage of some of these MAO-B inhibitor drugs gradually leads to many side effects such as toxicity, involuntary movement, malaise, or loss of efficacy. Therefore, it is important to discover novel MAO-B inhibitors when developing anti-PD drugs.

Herbal medicines have gained global recognition in the discovery of anti-Parkinson’s drugs. Several plant chemicals, such as caffeic acid, quercitrin, isoquercitrin, and kaempferol, have been identified to treat and manage Parkinson’s disease [15]. However, the complexity of herbal components, the tedious laboratory process, and the time taken by conventional isolation techniques make it difficult to identify the active components of crude drugs [16]. Recently, several proteins have been immobilized on different solid supports, such as the inner surface of the capillary electrophoresis tube, using polydopamine-coated magnetic microspheres to identify the ligands of proteins in herbal mixtures based on protein–ligand specific interactions [17,18,19]. Such a strategy, also called ligand fishing, has proved to be highly efficient in screening active natural products due to the high ligand–protein binding specificity, superparamagnetic properties, ease of protein immobilization, low sample consumption, and minimal pre-treatment complications [20,21]. Our group has applied ligand fishing based on magnetic nanoparticles to develop various rapid screening methods for identifying the ligands of target proteins from a variety of traditional Chinese medicinal plant extracts [22,23,24].

*Nigella glandulifera* Freyn et Sint. is an annual herbaceous plant belonging to the family of Ranunculaceae. This plant is a traditional Chinese herb used to treat and manage various ailments. The seeds are commonly consumed as an herbal tea and as food by the Tibetan and Xinjiang people of China, and have been documented ethnomedically to boost brain function, treat headaches, and manage many neurodegenerative diseases [25,26,27]. Among Chinese people, this plant is known as “Heizhongzicao” [28]. The seed is admirable in treating many diseases, such as respiratory, urogenital, and neurological diseases, as well as cancers, with little or no toxicity in the body [29]. Phytochemical studies on *N. glandulifera* seeds have reported the presence of several chemical components such as alkaloids, flavanol glycosides, phthalide derivatives, oleanane-type triterpenoid saponins, volatile oils, and fatty acids [30,31,32,33]. Pharmacological research has revealed that *N. glandulifera* seed extract induces antioxidant [33], anti-inflammatory [34], insulin-sensitizing [35], anti-tuberculosis [27], and anti-cancer [36,37] effects. Some saponins in the seeds attenuate collagen-induced rheumatoid arthritis [38], while volatile oils offer a neuroprotective effect by preserving mitochondrial metabolic enzyme activities and regulating the expression of apoptosis-related genes [39]. Nevertheless, very little is known about the chemical compounds responsible for the MAO-B inhibitory activity.

In this work, the MAO-B enzyme was immobilized on a functionalized magnetic nanoparticle to fish the enzyme inhibitors from the methanol extract of *N. glandulifera* seeds. The MAO-B inhibitory activity and neuroprotective potential on 6-hydroxydopamine (6-OHDA) induced PC-12 cells were tested. The inhibitory enzyme assay and molecular docking interaction were also investigated to evaluate the mechanisms of the interaction of the ligand with the identified MAO-B compounds.

## 2. Results and Discussion

### 2.1. Characterization of the Immobilized MAO-B

**Figure 1:**
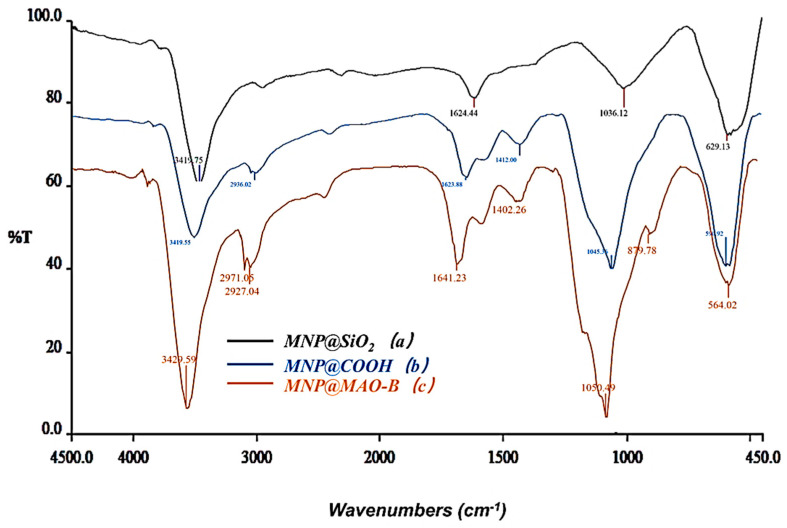
FT- IR spectra of (**a**) MNPs@SiO_2_; (**b**) MNPs@COOH; (**c**) MNPs@MAO-B.

Fourier transform infrared (FT-IR) spectrometry was used to characterize the MNPs@SiO_2_, MNPs@COOH, and MNPs@MAO-B, as shown in Figure 1. The absorption peak at 629 cm^−1^ in Figure 1 corresponds to the stretching vibration of the Fe-O bond in the MNPs. The peaks for MNPs@COOH (Figure 1) at 1624 cm^−1^ and 1412 cm^−1^ were due to the N-H bending and the C-O stretching vibration, respectively, indicating that the amino and carboxyl groups were successfully modified on the MNPs@SiO_2_. In the spectrum of MNP_S_@MAO-B (Figure 1), the absorption peaks at 1641 cm^−1^ and 1050 cm^−1^ are stronger compared to those in MNPs@COOH, which indicates that there was a significant increase in the amount of amino, carboxyl, and peptide bonds in the structure of MAO-B [40]. The results imply that MAO-B was successfully immobilized on the MNP surface. Moreover, the amount of MAO-B immobilized on MNPS@COOH was found to be 48 μg/mg using the Coomassie Brilliant Blue stain method and Bovine Serum Albumin (BSA) as a standard.

### 2.2. Identification of the Ligands of MAO-B

**Figure 2:**
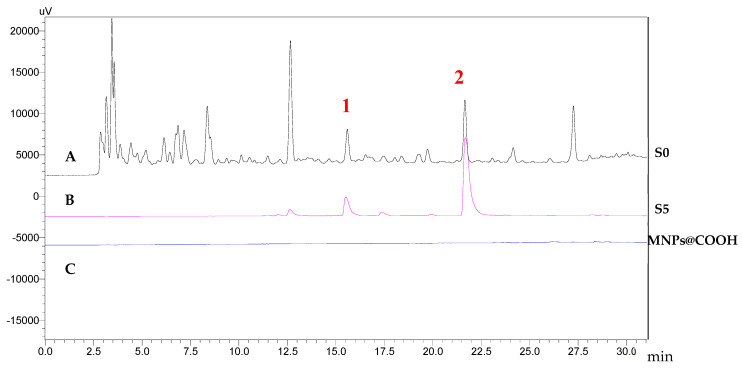
Chromatogram of S0 and S5 of *n*-BuOH fraction of *N. glandulifera* extract: (**A**) S0 refers to *n*-BuOH fraction; (**B**) S5 refers to components obtained from ligand fishing; (**C**) MNPs@COOH is the blank control for ligand fishing.

**Figure 3:**
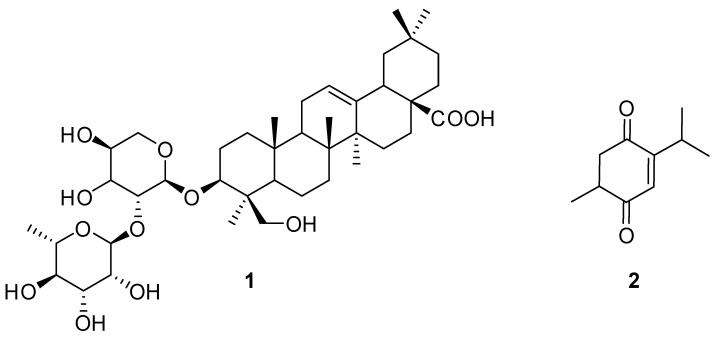
Identified chemical structures of MAO-B inhibitory compounds.

The bioactive ligands bound as MNPs@MAO-B from S0 were dissociated with 500 μL of 50% acetonitrile-water (ACN) and denoted as S5. The S0 showed several peaks, while the S5 showed only two corresponding major ligands in the chromatogram (Figure 2). Similar procedures and protocols have been successfully utilized on several MNP ligand fishing techniques, thus confirming the reliability of the magnetic nanoparticle solid phase extraction [41,42]. Furthermore, using MNPs@COOH as the blank control showed that no compound was observed in the corresponding HPLC-UV, confirming a non-specific binding on MNPs@MAO-B. The ligands were identified by HPLC-MS (Figures S1 and S2). The MS spectra revealed their molecular weights (Table S1). The molecular weight of ligand **1** was 750 (*m/z* 773, [M+Na]^+^). Ligand **2** had a molecular weight of 166 (*m*/*z* 167, [M+H]^+^). A comparison with the previously reported isolated compounds in the literature revealed that ligand **1** was tauroside E, and ligand **2** was thymoquinone [32,43]. The chemical structures of the two ligands are shown in Figure 3.

### 2.3. MAO-B Ligands from N. glandulifera

The preliminary MAO-B inhibitory assay showed that the inhibitory rates of both ligands (0.05 mg/mL) were above 50%. The half-maximal inhibitory concentration (IC_50_) values for ligands **1** and **2** were calculated to be 35.85 ± 0.03 µM and 25.54 ± 0.05 µM, respectively (Table 1). This is the first report on the MAO-B inhibitory activity of both compounds while supporting the ethnomedicinal claims of *N. glandulifera* as an anti-neurodegenerative herb and a functional food. Furthermore, previous studies report that several compounds from herbal medicines, such as caffeic acid, catechin, and esculin, possess significant MAO-B inhibitory effects [44,45,46,47]. Our findings support the conclusion of previous studies that medicinal plants are a rich source for discovering inhibitors of MAO-B [46,47].

### 2.4. Enzymatic Kinetic Study

**Figure 4:**
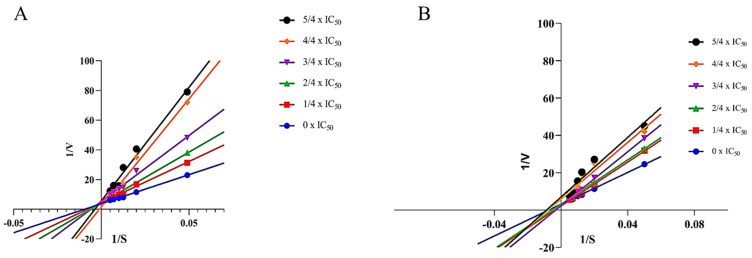
Kinetic inhibition using Lineweaver-Burk plots for MAO-B ligands **1** (**A**) and **2** (**B**).

The enzymatic kinetics of the enzyme inhibitors contribute additional information about the inhibitors’ specificity and effectiveness for drug development [48]. The enzymatic kinetics of ligands **1** and **2** were assayed using Lineweaver–Burk plots which were plotted by the inverse of the substrate concentrations and velocities in the presence of varying concentrations of the MAO-B inhibitor (0 × IC_50_, 1/4 IC_50_, 2/4 IC_50_, 3/4 IC_50_, 4/4 IC_50_, and 5/4 IC_50_). As shown in Figure 4A, six lines of Lineweaver–Burk plots of ligand **1** intersect the *y*-axis, revealing that ligand **1** is a competitive inhibitor. Competitive inhibitors compete with the substrate for the enzyme’s active site. On the other hand, six straight lines of ligand **2** intersect the *x*-axis, as shown in Figure 4B, indicating that ligand **2** inhibits MAO-B in a non-competitive manner, which means that it binds to a site other than the active site of the enzyme.

### 2.5. Molecular Docking

**Figure 5:**
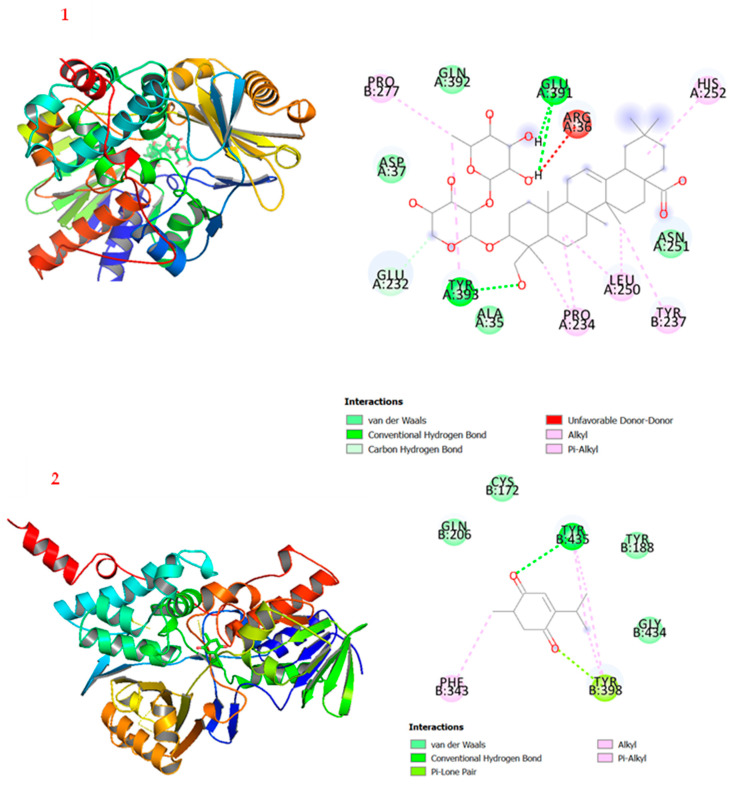
2D and 3D interactions of MAO-B with ligands **1** and **2**.

Molecular docking is a computer-aided technology used to evaluate potential drug candidates and attribute features of the receptor and the interaction based on the theoretical role of the ligands [49]. The 2D and 3D molecular docking simulations of the ligands to the enzyme are shown in Figure 5. The lowest binding affinity energies of **1** and **2** were −9.3 kcal/mol and −7.2 kcal/mol, respectively, compared with safinamide (−11.79 kcal/mol) [30]. It was observed in this study that compound **2** showed better MAO-B inhibitory activity compared with compound **1**, but the molecular docking investigation revealed that the affinity of compound **1** to the enzyme is stronger than that of compound **2**. Specifically, compound **1** showed two main conventional hydrogen bonds at Glu-391 and Tyr-393. Furthermore, there are alkyl and π-alkyl interactions between Pro-277, His-252, Leu-250, Tyr-237, and Pro-234 of the enzymes to the sugar moieties of compound **1**. As shown in Figure 5, compound **1** formed strong conventional hydrogen bonding with residues Glu-391 and Tyr-393, as well as alkyl and π–alkyl interactions with residues Pro-277, His-252, Leu-250, Tyr-237, and Pro-234. Similarly, compound **2** interacted with MAO-B by forming conventional hydrogen bonds with the residue of Tyr-435, as well as π–alkyl and alkyl interactions with the residue of Phe-343. These docking interactions showed some hypothetical evidence about the role of these compounds as MAO-B inhibitors and their possible contribution in treating and managing Parkinson’s disease.

### 2.6. Neuroprotective Effect of the Inhibitors on 6-OHDA-Induced PC-12 Cells

**Figure 6:**
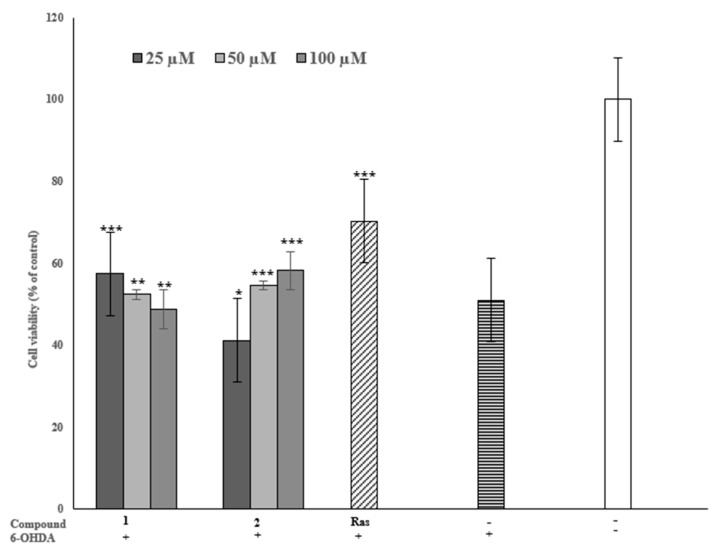
Neuroprotective effects of inhibitors on 6-OHDA-induced PC-12 cell injury. Ras (Rasagiline) was used as a positive control. Data (cell viability) are expressed as mean ± SD (* *p <* 0.05, ** *p <* 0.01, and *** *p <* 0.001 compared with the model group).

The 6-OHDA neurotoxin is a widely utilized method of studying the therapeutic potential of drugs to treat PD. Since 6-OHDA shows the ability to cause the degeneration of dopaminergic neurons, and pheochromocytoma-12 (PC-12) cells injured by 6-OHDA showed a PD-like cellular model for the screening of anti-PD compounds, we assayed the neuroprotective effect of the identified inhibitors against 6-OHDA-induced PC-12 cells. The concentration of 6-OHDA was stabilized at 300 μM by comparing the damage degree to the cells caused by a series of concentrations (25 μM, 50 μM, and 100 μM). Compared to the PD-like model cells, the viability of cells pre-treated with ligand **1** at 25 μM increased from 50% to 58% and was statistically significant (*p <* 0.001); at 50 μM, it showed a slightly protective effect of 52% (*p <* 0.01); and at 100 µM, there was a decrease *(p <* 0.05) in the neuroprotective effect. Notably, ligand 2 (50 µM) showed significantly (*p <* 0.001) increased neuroprotection compared with the model PC-12 cells from 50% to 55%, and the 100 µM concentration was 58% (Figure 6). It was noticed that ligand **2** offered more neuroprotection than ligand **1** and that the higher the concentration, the stronger the neuroprotection. On the other hand, ligand **1** might have cell toxicity at high concentrations. These findings show that the two inhibitors offer a dose–dependent protective effect against 6-OHDA-induced PC-12 cells. The neuroprotective roles of some neuroactive components against 6-hydroxydopamine-induced PC-12 cells have previously been reported towards the development of Parkinson’s disease drugs [40,50].

## 3. Materials and Methods

### 3.1. Materials, Reagents, and Instruments

Chemicals such as sodium hydroxide (NaOH), 3-aminopropyl-trimethoxysilane (APTMS), and dimethyl sulfoxide (DMSO) were obtained from the Chengdu Kelong chemical reagent factory (Chengdu, China). Monoamine oxidase B (MAO-B, 100.23 U/mL) was prepared in-house [12]. Kynuramine dihydrobromide was purchased from Sigma-Aldrich (St Louis, MO, USA). Safinamide mesylate and rasagiline were purchased from Meilunbio (Dalian, China). The MAO-B inhibition assay was carried out using Thermo Scientific Varioskan Flash equipped with a 96-well microplate (Thermo, Waltham, MA, USA). FT-IR spectra were recorded in KBr with a PerkinElmer FT-IR spectroscope (PerkinElmer, Waltham, MA, USA). Ultrapure water produced with a UP water purification (18.25 MΩ) system (Ultrapure, Chengdu, China) was used for HPLC. HPLC-grade methanol was obtained from JT Baker (Phillipsburg, NJ, USA). The HPLC system consisted of a Shimadzu LC-20 AD series equipped with a thermostatic column compartment, an SPD20A UV-vis detector (Shimadzu, Kyoto, Japan), and an Agilent ZORBAX SB-C18 column (4.6 × 250 mm, 5 μm). The mobile phase was composed of solvent A (0.1%, *v*/*v*, formic acid/H_2_O) and solvent B (100% MeOH) at a flow rate of 0.8 mL/min, and an injection volume of 20 µL. The eluting gradient was set to 30–100% MeOH for 0–30 min. The MS detection was in the positive ion mode using a capillary voltage of 3.0 kV; a source temperature of 180 °C; a desolvation temperature of 350 °C; and a desolvation gas flow of 800 L/h, while the nebulizer was set at 0.8 Bar and the sample flow rate was set at 0.3 mL/min for the ESI-MS (Bruker Compass Data Analysis 4.0; microTOF-Q11-10203). For the FT-IR, the samples were pretreated with a tablet press using potassium bromide (KBr) in a dry environment, and the wavenumbers of the FT-IR measurement were set in the range of 450 to 4500 cm^−1^.

### 3.2. Extraction of Seeds of N. glandulifera

*N. glandulifera* dried seeds were purchased from a local drug market in Lhasa, Tibet Autonomous Region of China. The whole plant, including the seeds, was identified by Professor Yong-Mei Zhang at the Chengdu Institute of Biology at the Chinese Academy of Sciences. A voucher specimen (2020-05) was deposited at the same institute. The seeds were pulverized and extracted with 80% aqueous MeOH under ultrasonication for 30 min. The organic solvent was removed to yield an aqueous solution, which was partitioned successively by petroleum ether (PE), ethyl acetate (EtOAc), and *n*-butanol (*n*-BuOH) to obtain the respective fractions.

### 3.3. Preparation and Characterization of MAO-B Functionalized Magnetic Nanoparticles

The MAO-B inhibitory activity of the two isolated ligands was tested according to the previous procedure with minor modifications [22]. In brief, 2.0271 g of FeCl_3_·6H_2_O and 0.7407 g of FeCl_2_·4H_2_O in 1:2 molar ratios were dissolved in 250 mL H_2_O, and ammonia water was added to adjust the pH to 9–10 before stirring under a nitrogen atmosphere for approximately 30 min at room temperature. An external magnet was used to separate the magnetic nanoparticles (MNPs), which were consecutively washed with water and ethanol. The MNPs were re-suspended in 150 mL of ethanol containing 400 µL of tetraethyl orthosilicate (TEOS), followed with the addition of ammonia water to adjust the pH to 9–10 and stirred for 5 h to produce a core-shell structure of MNPs@SiO_2_. The latter was separated using an external magnet, washed with water and ethanol, and then mixed with 2 mL of 3-aminopropyltriethoxysilane (APTMS) in 90 mL ethanol containing 1 mL water at 35 °C to obtain the amino-terminated MNPs (MNPs@NH_2_). Then, 500 mg of lyophilized MNPs@NH_2_ was treated with 3 g of succinic anhydride in 30 mL of dimethyl formamide (DMF) to terminate the MNPs with carboxyl groups as MNPs@COOH. Subsequently, 3 mg of MNPs@COOH was dispersed in 3 mL of MES buffer (PBS 50 mM, pH 7.4) containing 10 mM EDC·HCL and 20 mM NHS, and vortexed for 30 min, then MAO-B (2.5 U/mL) was added and incubated for 24 h at 25 °C. Finally, the obtained MNPs immobilizing the MAO-B (MNPs@MAO-B) were separated by a magnet and washed three times with PBS (50 mM, pH 7.4) to dissociate the unbound enzyme. To confirm the functionality of the synthesized magnetic nanoparticles, a Fourier transform infrared (FT-IR) spectrometer was used to characterize MNPs@SiO_2_, MNPs@COOH, and MNPs@MAO-B, as previously described in our laboratory.

### 3.4. Ligand Fishing of N. glandulifera

The *n*-BuOH fraction of the *N. glandulifera* extract was used for the ligand fishing test due to its moderate MAO-B inhibitory activity. First, the *n*-BuOH fraction was filtered with a 0.22 µm filtration membrane, concentrated to dryness, and diluted in PBS (50 mM, pH 7.4) to a concentration of 1 mg/mL, denoted as S0. A total of 3 mL of S0 was added to a 5 mL Eppendorf tube containing 20 mg of MNPs@MAO-B. The tube was oscillated for 30 min at room temperature, and the MNPs@MAO-B adsorbed with ligands of the enzyme were separated by an external magnet and washed three times using PBS (50 mM, pH 7.4). Then, 500 µL of 50% ACN was used to dissociate the ligands bound to the MNPs@MAO-B, denoted as S5. The S0 and S5 were analyzed by HPLC to determine the possible ligands of the enzyme.

### 3.5. Isolation of the Target Ligands

The two peaks that appeared in the high-performance liquid chromatography (HPLC) chromatogram of S5 were noted to be ligands of the enzyme. Under the guidance of HPLC, the *n*-BuOH fraction was subjected to column chromatography on ODS (MeOH/H_2_O, 20:80, 50:50, and 100:0, *v*/*v*), silica gel (CH_2_Cl_2_/MeOH, from 100:1 to 1:1, *v*/*v*), and Sephadex LH-20 (MeOH 100%, *v*/*v*) to afford the two compounds (Figure S3).

### 3.6. Monoamine Oxidase B Inhibition Assay

The MAO-B inhibitory activity of the two isolated ligands was tested according to the previous procedure with minor modifications [21]. First, 50 μL of MAO-B (2.5 U/mL) was incubated with 100 μL of the test compound (inhibitor) in a 96-well ELISA plate at 37 °C for 10 min. Secondly, 50 μL of kynuramine (50 µM) was added to incubate at 37 °C for 30 min before 80 μL of NaOH (2N) was introduced to end the reaction. The absorbance was read using a multimode microplate reader (Varioskan Flash, 310/400 ƛex/em). Meanwhile, 0.01 mg/mL of safinamide, a known MAO-B inhibitor for the treatment of PD, was used as a positive control. Assays were performed in triplicates. Data were analyzed using GraphPad Prism 6.0 software and expressed as the mean ± standard deviation. The percentage inhibition and the half-maximal inhibitory (IC_50_) values of the active compounds for MAO-B were determined using Ellman’s method [51]:

(1) $$\mathrm{Inhibition}\;\mathrm{rate}\;(\%)=\frac{B0-\left(B1-B2\right)}{B0}\times 100$$

where *B*0 represents the absorbances of the test blank (PBS and MAO-B), *B*1 represents the absorbances of the sample, and *B*2 represents the absorbances of the control blank (PBS, MAO-B, and Kynuramine).

### 3.7. Enzymatic Kinetic Study

The Lineweaver–Burk plot was used for the kinetic assay, the concentrations of kynuramine were between 20 and 180 μM, and the different folds of the ligands were prepared based on the IC_50_. The concentration of the MAO-B used was 2.5 U/mL, and the reaction was monitored using a plate reader after 10 min.

The Lineaweaver–Burk plot was calculated as:

(2) $$\frac{\left[S\right]}{V}=\frac{Km}{V_{\max}}+\frac{\left[S\right]}{V_{\max}}$$

where [*S*] is the concentration of MAO-B, and *v* and *V_max_* represent the enzyme reaction rate and the maximum enzymatic reaction velocity, respectively.

### 3.8. Molecular Docking

Molecular docking was optimized to predict the optimal binding mode of the ligand–receptor complex by studying the binding affinity and the amino acid residue environments [52]. The MAO-B protein (PDB ID: 2V5Z) was retrieved from (http://www.rcsb.org/pdb/accessed on 10 September 2022) and the three-dimensional (3D) structure of MAO-B (*Homo sapiens*) was downloaded from the Protein Data Bank (http://www.rcsb.org/pdb/home/home.do accessed on 10 September 2022). The ligands were drawn with ChemBio-Draw Ultra 14.0, while the protein and compounds were converted to PDBQT (Protein Data Bank, Partial Charge (Q) and Atom Type (T)) files, and the docking input files were generated using AutoDock Tools 1.5.6 (Scripps Research, 211 San Diego, CA, USA). The genetic algorithm (GA) was set up 1000 times to run the hit, and the value of exhaustiveness was set to 27. The search grid was set up at center x = 50.757, y = 155.963, and z = 27.636, and the dimension sizes were set up at x = 31.58, size y = 35.50, and size z = 30.066. Gasteiger charges and the number of torsions were set for metabolites, and polar hydrogens were merged. For the receptor polar hydrogens, Kollman charges were added, solvation parameters were assigned by default, and for Vina docking, the default parameters were used. The binding energy predicted by AutoDock Vina with the lowest energy mode was selected as the best binding energy affinity and the output files were visualized using Discovery Studio and PyMol.

### 3.9. Protective Effect of the Ligands on 6-OHDA-Induced PC-12

The PC-12 cells were obtained from Hunan Fenghui Biotechnology Co., Ltd. (Changsha, China). The 6-hydroxydopamine (6-OHDA) was used because it is a toxic oxidative metabolite of dopamine and has been used effectively to evaluate potential anti-Parkinsonism agents [53]. The cells were cultured in DMEM supplemented with 5% FBS and 1% penicillin-streptomycin, and maintained at 37 °C with 5% CO_2_. The cells were trypsinized when they achieved over 90% growth on the bottom and seeded in 96-well microplates (1 × 10^4^ cells/well) to incubate in the same medium and environment as above. After being cultured for 24 h, PC-12 cells were treated with 100 μL of each test compound at various concentrations (25 μM, 50 μM and 100 μM) for 2 h, and then the cells were incubated with 6-OHDA for another 24 h. At the same time, rasagiline was used as the positive control in this experiment because safinamide failed to exhibit significant protective effects on PC-12 cells as rasagiline. The optical densities were measured at 450 nm using a multimode microplate reader (Thermo Fisher, Waltham, MA, USA).

### 3.10. Data Analysis

Data were expressed as the mean ± standard deviation (SD) from three replicate experiments. GraphPad Prism 6.0 was used as the statistical analysis software. The one-way analysis of variance (ANOVA) and the mean ± standard deviation (*n* = 3) were separated. The *p* < 0.05 was considered to be statistically significant. Finally, ChemDraw version 14 (student version) was used to draw the chemical structures in this study.

## 4. Conclusions

This study identified two MAO-B inhibitors (tauroside E and thymoquinone) from *N. glandulifera* dried seeds using a ligand fishing technique based on MAO-B functionalized magnetic nanoparticles. In this work, the in vitro MAO-B inhibition activity and neuroprotective potential on 6-OHDA-induced PC-12 cells of both ligands were reported for the first time, with IC_50_ values of 35.85 μM and 25.54 μM, respectively. Both compounds exhibited neuroprotective effects on 6-OHDA-induced PC-12 cells by increasing viability by 52% and 58%, respectively. The molecular docking interactions revealed that these inhibitors might play significant roles in neuroactive ligand–receptor interactions in the major neurodegenerative pathway and PD. The findings contribute to the fast screening of neuroprotective components of *N. glandulifera* using magnetic nanoparticle ligand fishing, and justify its health benefits as a food and an herb in managing PD and other neurological diseases.

## Figures and Tables

**Table 1:** Inhibitory effects of compounds **1** and **2** on MAO-B.

Compound	Inhibitory MAO-B (100 µM) %	IC_50_ Inhibitory Concentration µM	R^2^
*n*-BuOH fraction	54.80	-	-
1	55.80	35.85 ± 0.03	0.98
2	65.70	25.54 ± 0.05	0.99
Positive control (Safinamide)	98.50	0.19 ± 0.09	0.99

## Data Availability

Not applicable.

## Supplementary Materials

The following supporting information can be downloaded at: https://www.mdpi.com/article/10.3390/plants12040882/s1, Table S1: ESI-MS of MAO-B ligands isolated; Figure S1: ESI-MS of compound 1; Figure S2: ESI-MS of compound 2; Figure S3: Chromatogram of the S5 and the isolated compounds **1** and **2**.
