# Supplementary material for: Screening of Monoamine Oxidase Inhibitors from Seeds of Nigella glandulifera Freyn et Sint. by Ligand Fishing and Their Neuroprotective Activity
Source: Plants (Basel). 2023 Feb 15;12(4):882. doi: 10.3390/plants12040882 (PMC9960078; doi:10.3390/plants12040882)
Supplement: Supplementary file 1 [file plants-12-00882-s001.zip › plants-2190806-supplementary.pdf]

plants

SUPPLEMENTARY MATERIALS

Screening of Monoamine Oxidase Inhibitors from Seeds of *Nigella glandulifera* Freyn et Sint. by Ligand Fishing and Their Neuroprotective Activity

Emmanuel Ayodeji Ayeni [1,2], Chao Ma [3], Yi-Kao Hu [1,2], Xiao-Lin Bai [1,2], Yong-Mei Zhang [1,*] and Xun Liao [1,*]

[1] Chengdu Institute of Biology, Chinese Academy of Sciences, Chengdu 610041, China
[2] University of Chinese Academy of Sciences, Beijing 100049, China
[3] Phytochemistry Laboratory, Tibet Plateau Institute of Biology, Lhasa 850001, China
* Correspondence: zhangym@cib.ac.cn (Y.-M.Z.); liaoxun@cib.ac.cn (X.L.); Tel.: +028-82890756 (Y.-M.Z.); Tel.: +86-18010696109 (X.L)

Abstract: *Nigella glandulifera* is a traditional medicinal plant used to treat seizures, insomnia, and mental disorders among the Tibetan and Xinjiang people of China. Recent pharmacological research indicates that the seeds of this plant have a neuroprotective effect; however, the chemical components responsible for this effect are unknown. Monoamine oxidase B (MAO−B) has been recognized as a target for developing anti-Parkinson's disease drugs. In this work, MAO−B functionalized magnetic nanoparticles were used to enrich the enzyme's ligands in extracts of *N. glandulifera* seeds for rapid screening of MAO−B inhibitors coupled with HPLC−MS. Tauroside E and thymoquinone were found to inhibit the enzyme with IC_{50} values of 35.85 μM and 25.54 μM, respectively. Both compounds exhibited neuroprotective effects on 6−OHDA-induced PC−12 cells by increasing the cell viability to 52% and 58%, respectively, compared to 50% of the injured cells. Finally, molecular docking indicated strong interactions of both inhibitors with the enzyme. This work shows that MAO−B functionalized magnetic nanoparticles are effective for rapid screening of anti-PD inhibitors from complex herbal mixtures and, at the same time, shows the promising potential of this plant's seeds in developing anti−PD drugs.

Table S1. ESI−MS of MAO-B ligands isolated.

Figure S1. ESI−MS of compounds **1**.

Figure S2. ESI−MS of compounds **2.**

Figure S3. Chromatogram of the S5 and the isolated compounds **1** and **2**

Table S1. ESI−MS of MAO-B ligands isolated

Compound	Pseudo molecular peak	Mass (m/z)	Error (ppm)	Calculated mass	Molecular formular	Compound identified
**1**	$[M+Na]^+$	773.44	3.6	750.44	$C_{41}H_{66}O_{12}$	Tauroside E
**2**	$[M+H]^+$	167.08	-20.2	166.08	$C_{10}H_{12}O_2$	Thymoquinone

Mass Spectrum SmartFormula Report

Analysis Info

Analysis Name	D:\data\USER-2022\NG-1.d	Acquisition Date	6/2/2022 3:13:52 PM
Method	tune_low_NEW.m	Operator	Ma
Sample Name	NG-1	Instrument / Ser#	micrOTOF-Q II 10203
Comment			

Acquisition Parameter

Source Type	ESI	Ion Polarity	Positive	Set Nebulizer	0.8 Bar
Focus	Not active	Set Capillary	4500 V	Set Dry Heater	180 °C
Scan Begin	50 m/z	Set End Plate Offset	-500 V	Set Dry Gas	6.0 l/min
Scan End	1200 m/z	Set Collision Cell RF	150.0 Vpp	Set Divert Valve	Source

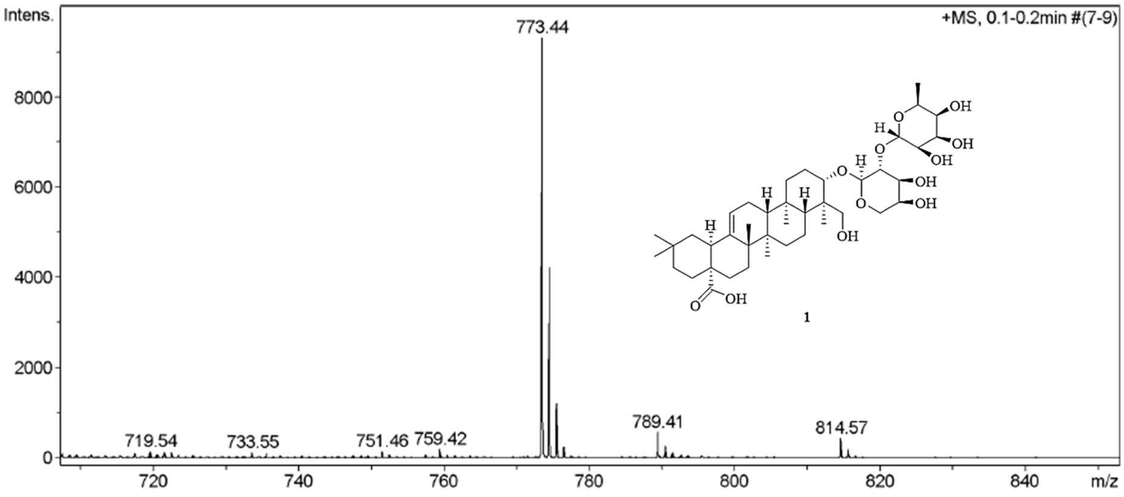

Meas. m/z	Formula	m/z	err [ppm]	mSigma	N-Rule	e⁻ Conf
773.44						
	C 41 H 66 Na O 12	773.44	3.6	2.23	ok	even

Figure S1. ESI−MS of compound **1**

Mass Spectrum SmartFormula Report

Analysis Info

Analysis Name	D:\data\USER-2022\NG-2.d	Acquisition Date	6/2/2022 3:15:08 PM
Method	tune_low_NEW.m	Operator	Ma
Sample Name	NG-2	Instrument / Ser#	micrOTOF-Q II 10203
Comment			

Acquisition Parameter

Source Type	ESI	Ion Polarity	Positive	Set Nebulizer	0.8 Bar
Focus	Not active	Set Capillary	4500 V	Set Dry Heater	180 °C
Scan Begin	50 m/z	Set End Plate Offset	-500 V	Set Dry Gas	6.0 l/min
Scan End	1200 m/z	Set Collision Cell RF	150.0 Vpp	Set Divert Valve	Source

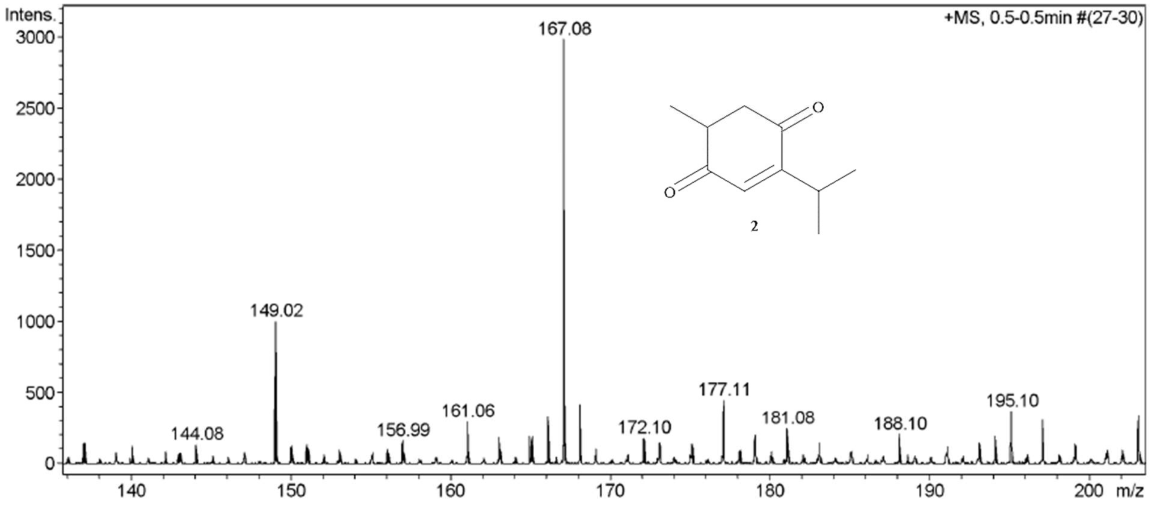

Meas. m/z	Formula	m/z	err [ppm]	mSigma	N-Rule	e⁻ Conf
167.08						
	C 13 H 11	167.09	3.9	14.91	ok	even
	C 8 H 11 N 2 O 2	167.08	-20.2	31.26	ok	even

Figure S2. ESI–MS of compound **2**

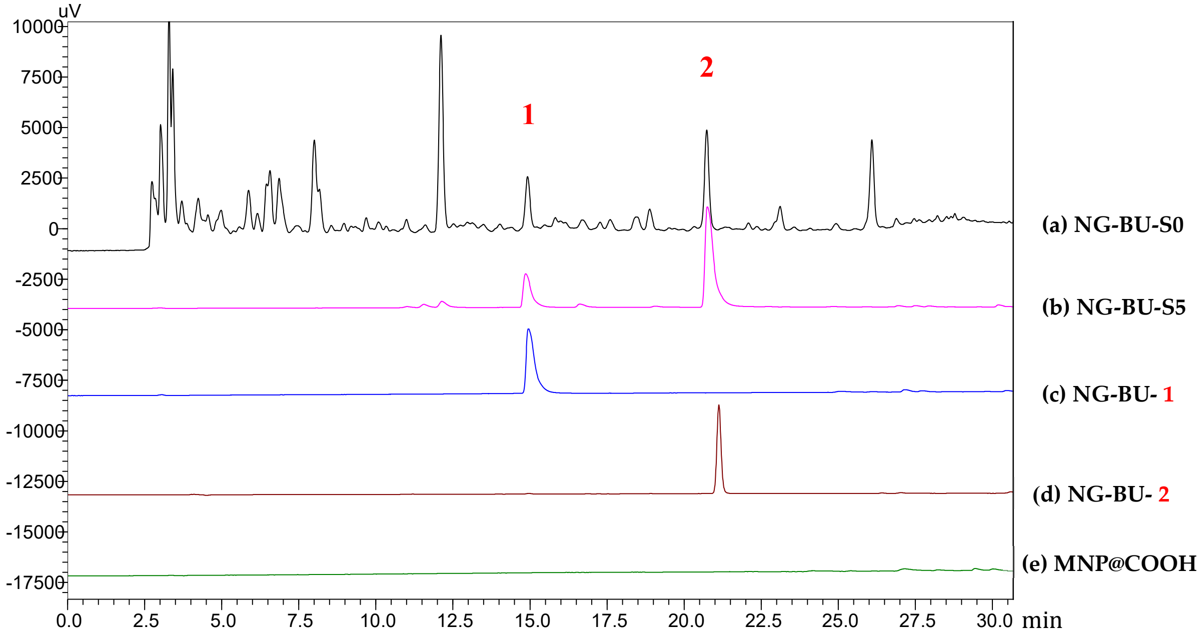

Figure S3. Chromatogram of the S5 and the isolated compound **1** and **2**
